# Supplementary material for: State of the Art of the Molecular Biology of the Interaction between Cocoa and Witches’ Broom Disease: A Systematic Review
Source: Int J Mol Sci. 2023 Mar 16;24(6):5684. doi: 10.3390/ijms24065684 (PMC10057015; doi:10.3390/ijms24065684)
Supplement: Supplementary file 1 [file ijms-24-05684-s001.zip › Supplementary File S1. PRISMA_checklist .pdf]

PRISMA 2020 Checklist

Section and Topic	Item #	Checklist item	Location where item is reported
**TITLE**			
Title	1	The state of the art of the molecular biology of the interaction between cocoa and witches' broom disease.	Title page, Page# 1
**ABSTRACT**			
Abstract	2	Witches' broom disease (WBD) caused by the hemibiotrophic fungus *Moniliophthora perniciosa* is one of the most important socio-economic phytosanitary problems of cocoa crops in the Americas. Scientific advances towards the elucidation of this pathosystem have occurred in recent years, but the Molecular Biology understanding of this pathogen-host interaction is still a field with many unanswered questions. In order to gain new insights and understand WBD at a molecular level, we present the first systematic review on the topic. Scopus, Web of Science, Pubmed and Scielo databases were used. A total of 1118 studies were extracted from the databases. Of these, 109 were eligible for data summarization and to answer the scientific questions of the review, using the inclusion and exclusion criteria, based on the Prisma guidelines. Eligible studies show that understanding the transition from the biotrophic-necrotrophic phase of the fungus is crucial for disease control. In recent years, protein profiles of *M. perniciosa* have been traced and some of the proteins have great biotechnological potential or can be targets for intervention, but tests in this regard are still lacking. Eligible articles in this study also revealed the potential genes in the interaction of *M. perniciosa* and hosts and Molecular Markers more efficient in the search for genetic variability and source of resistance. We highlight an arsenal of effectors already identified and not explored in the *M. perniciosa* x hosts pathosystem. This Systematic Review contributes to the understanding of the Molecular Biology of *M. perniciosa* and its interaction with the hosts, in addition, it offers new insights in this field of study and proposes different paths for the development of new strategies to control WBD.	Abstract, Page# 1
**INTRODUCTION**			
Rationale	3	Chocolate or cupulate, the main products from plant species of the genus Theobroma, are threatened by the witches' broom disease (WBD) caused by *Moniliophthora perniciosa*, a devastating fungus that compromised the production of these products in the years 2017 to 2020 in 190 thousand tons. (ICCO, 2022). Brazil was the largest producer, with the arrival of the WBD, production declined and its position in the world ranking of cocoa bean production did not recover, currently occupying the seventh position (FAOSTAT, 2020). Existing information that assembles this biological puzzle of the pathosystem of *M. perniciosa* has never been systematized to the point of understanding the molecular biology of the fungus and its mechanism of action. Despite this significant advance, many questions about the molecular mechanisms of control and change remain unsolved - at the experimental level - and remain until the development of this study. The collection of knowledge on Genetics, Structural Genomics, Molecular Mechanisms of Action of the fungus *M. perniciosa*, and host defense, together with information on study strategies, main research hotspots and tools used in the fungus infection strategy are important gaps that must be completed in order to contribute to the elucidation of the pathosystem of *M. perniciosa* and, consequently, to the genetic improvement.	Introduction Page #2
Objectives	4	This is the first systematic review on the subject, which aims to demonstrate the state of the art of the molecular biology of the witches' broom disease caused by *M. perniciosa*	Introduction Page #2
**METHODS**			
Eligibility criteria	5	Scientific articles indexed in peer review journals in english.	Methods Page #28 - #30
Information sources	6	The research was carried out using previously selected databases such as: Pubmed, Scopus, Scielo and Web Of Science. The final date for accessing the information was in March 2022	Methods Page #28 - #30

PRISMA 2020 Checklist

Section and Topic	Item #	Checklist item	Location where item is reported
Search strategy	7	The search strategies were based on the development of search strings and Boolean connectors were used to make the searches expressive, in addition to specifying searches by titles and abstracts in the database.	Methods Page #28 - #30
Selection process	8	The methods used to decide whether a study met the review's inclusion criteria were based on first reading titles and abstracts using the Start software and then reading the full paper.	Methods Page #28 - #30
Data collection process	9	The collected data were shared and distributed among the reviewers, using a dynamic Excel spreadsheet, the Start program and the statistical environment of the R program.	Methods Page #28 - #30
Data items	10a	- Research groups/Research Centers/Laboratories and/or Universities that develop the studies;<br>- Study countries;<br>- Area of knowledge of publications;<br>- Hosts of the fungus;<br>- Molecular mechanisms induced in the fungus;<br>- Molecular mechanisms induced in the hosts;<br>- Genes related to resistance or susceptibility (hosts);<br>- Genes related to virulence (fungus);<br>- Sources of resistance developed (hosts);<br>- Molecular resistance markers (host);<br>- Genes expressed in the fungus x hosts interaction;<br>- Proteins accumulated in the fungus x hosts interaction;<br>- Biological function of genes and/or proteins expressed in the fungus x hosts interaction;<br>- Epigenetic mechanisms involved in resistance or susceptibility (hosts);<br>- Morphological changes in the fungus in the infection;<br>- Morphological changes of hosts when infected.	ND
	10b	Supposition made about any missing or unclear information in the collected studies were based on other studies that were not collected on the topic.	Discussion Page #14 - #27
Study risk of bias assessment	11	We follow the inclusion and exclusion criteria and also adopt the PICO strategy and PRISMA guidelines	Methods Page #2 and #3
Effect measures	12	No meta-analysis was applied in the study	ND
Synthesis methods	13a	The processes used to decide which studies were eligible are listed in item #5	Methods Page #28 - #30
	13b	The data are presented in full from their respective studies, no statistical treatment of the collected data was used.	Methods Page #28 - #30
	13c	The data were tabulated in dynamic Excel spreadsheets for further summarization.	ND
	13d	No meta-analysis was applied in the study	ND

PRISMA 2020 Checklist

Section and Topic	Item #	Checklist item	Location where item is reported
	13e	No subgroup analysis or meta-regression was applied	ND
	13f	Sensitivity analyzes were not applied to the data	ND
Reporting bias assessment	14	In addition to the pre-established criteria, item #11, we also analyzed the methodology applied in the studies. We analyzed whether the experimental designs were able to achieve the results obtained in the study. Example: Presence of control, number of repetitions, statistical analysis, incubation time, etc.	Discussion Page #14 - #27
Certainty assessment	15	We did not use any method to assess certainty, only the pre-established criteria item #11 and #14 were followed.	Discussion Page #14 - #27
**RESULTS**			
Study selection	16a	The results of the search and selection process are represented by a flowchart following the PRISMA guidelines.	Results Page #4-13
	16b	Literature review studies met some inclusion criteria but were excluded as they were not primary studies	Results Page #4-13
Study characteristics	17	The research questions were applied to all selected articles to extract the information that underlies the review.	Results Page #4-13
Risk of bias in studies	18	No items were selected outside the exclusion criteria.	Results Page #4-13
Results of individual studies	19	The individual results that answered the Systematic Review questions were systematized and summarized in tables and figures.	Results Page #4-13
Results of syntheses	20a	The results were summarized in figures and tables.	Results Page #4-13
	20b	We do not employ meta-analysis	ND
	20c	As we did not have a meta-analysis, we did not perform a heterogeneity analysis.	ND
	20d	As we did not have a meta-analysis, we did not perform a sensitivity analysis.	ND
Reporting biases	21	In order to reduce the risk of bias, we chose to insert only articles with scientific and statistical data and also those that really considered our main and secondary questions whose conclusions were reliable.	ND
Certainty of evidence	22	Results presented are based on primary studies selected based on inclusion criteria.	Results Page #4-13
**DISCUSSION**			
Discussion	23a	Brazil leads the production of knowledge on *Moniliophthora perniciosa*<br>The peculiar battle of a hemibiotrophic fungus and its hosts<br>Structural genomics of the causal agent of witches' broom<br>The hidden biotechnological potential of *M. perniciosa* pathosystem proteins	Discussion Page #14 - #27

PRISMA 2020 Checklist

Section and Topic	Item #	Checklist item	Location where item is reported
	23b	There were no limitations to the evidence included in the review. Evidence is based on experimental studies that were eligible for review because they met the inclusion criteria as described in items #5 #8 and #14	ND
	23c	All the steps performed in the Systematic Review were well established after arduous team discussions and, added to the use of Start and R studio software, reduced any limitations in the review processes.	Discussion Page #14 - #27
	23d	This review highlights considerable information accumulated in recent years and sheds light on the data published through systematization, allowing the understanding of the Molecular Biology of *M. perniciosa* and the identification of gaps in the study strategies already used. Although in recent years invaluable methods and resources have been developed to understand the Molecular Biology of *M. perniciosa*, along with its interaction with hosts, it is still important to determine how the biotrophic phase is maintained for a long time in M. perniciosa and how at the molecular level their hosts contribute to the end of this phase of WBD. Understanding the transition of this fungal phase is crucial for the control of the disease, as well as, for the development of resistant hosts, perhaps developing methods such as the CRISPR system, with the Knockout of genes of interest or using Epigenetic mechanisms will bring us understanding of the main genes involved in the end of this fungal stage and how to manipulate them. Most of the proteins identified in the different studies have great biotechnological potential, mainly the fungal effectors, still poorly characterized functionally, but which are crucial in the molecular battle *M. perniciosa* x hosts. It is estimable that the search for this understanding must continue, however, this study presents to the scientific community which pieces of this biological puzzle are still missing, and the prospects for achieving them, as we also present genes, proteins, molecular markers, physiological effects and biochemicals already massively identified.	Discussion Page #14 - #27
**OTHER INFORMATION**			
Registration and protocol	24a	The protocol was not previously registered, however it was discussed and evaluated in a team.	ND
	24b	The protocol can be accessed at: https://github.com/ArianaSantos/Santos-et-al.2022_systematic-review.git	Methods Page #28 - #30
	24c	No protocol information has been changed.	ND
Support	25	The authors are thankful for the grants and support from Coordenação de Aperfeiçoamento de Pessoal de Nível Superior - CAPES code 001 and Programa Nacional de Pós-Doutorado – PNPD, Brazil.The authors are thankful for the grants and support from Concelho Nacional de Desenvolvimento Científico e Tecnologia – CNPq code Desenvolvimento Tecnológico Industrial – DTI A, Brazil.	Page #30
Competing interests	26	The authors declare no competing interests.	Page #30
Availability of data, code and other materials	27	All data is available in the database: https://github.com/ArianaSantos/Santos-et-al.2022_systematic-review.git	Methods Page #28 - #30

From: Page MJ, McKenzie JE, Bossuyt PM, Boutron I, Hoffmann TC, Mulrow CD, et al. The PRISMA 2020 statement: an updated guideline for reporting systematic reviews. BMJ 2021;372:n71. doi: 10.1136/bmj.n71

For more information, visit: http://www.prisma-statement.org/
