# Supplementary material for: State of the Art of the Molecular Biology of the Interaction between Cocoa and Witches’ Broom Disease: A Systematic Review
Source: Int J Mol Sci. 2023 Mar 16;24(6):5684. doi: 10.3390/ijms24065684 (PMC10057015; doi:10.3390/ijms24065684)
Supplement: Supplementary file 1 [file ijms-24-05684-s001.zip › Supplementary Table S2.pdf]

Supplementary Table S2: Host molecular markers summarized from eligible studies in the systematic review.

Host	Molecular Marker						
	**Class**	**Name**	**Function**	**Region**	**Position (cM)**	**Genotype**	**Authors**
Theobroma cacao L. .	SSR	mTcCIR21	Resistance WBD	Nuclear	24	TSH 1188 CCN 51	Santos et al. 2007
		mTcCIR24			31		
		mTcCIR29			20		
		mTcCIR30			28		
		mTcCIR33			13		
		mTcCIR35			23		
	RAPD	r AV14.940	Resistance WBD	NA	~8,7	F2 (Sca-6 x ICS-1)	Queiroz et al 2003
	EST-SSR	msEstTsh-1	Resistance WBD	ND	NA	F2 (Sca-6 x ICS-1)	Lima et al. 2008
		msEstTsh-2		5′ UTR			
		msEstTsh-3		ORF			
		msEstTsh-4		ORF			
		msEstTsh-5		ORF			
		msEstTsh-6		ND			
		msEstTsh-7		ND			
		msEstTsh-8		ORF			
		msEstTsh-9		ND			
		msEstTsh-10		5' UTR			
		msEstTsh-11		3' UTR			
	SNP	NA	Deformed branches WBD	NA	9 e 29	Criollo	Osorio-Guarín et al. 2020
			Flower cushion broom WBD		1; 770 e 4		
			Harvested healthy pods		10 e 21		
			Deformed branches WBD		9 e 27	Matina	
			Flower cushion broom WBD		1; 794 e 4		
			Harvested healthy pods		9; 8 e 9		
	SSR	Y16983	Resistance WBD	Nuclear	NA	FO37-09 FO36-11 FO85-09 FO32-10 FO36-10 FO35-09 FO51-06 FO29-09	Lima et al. 2013
		Y16987					
		AJ271945					
		Y16 883					
		Y16984					
		AJ271827					
		AJ271944					
		AJ271946					
		AJ271953					
		Y16980					
		AJ271956					
		Y16981					
		Y16978					

GWAS-SNP	NA	Chirimoya Pod	Cromossomo I; II; VI e VII	NA	Progênies (Wild types x Known clones x Nacional)	McElrot et al. 2018
		Cushion Broom	Cromossomo I; VII e X			
		Vegetative Broom	Cromossomo VIII E IX			
SNP	Tcm003s33466269	Resistance WBD	Cromossomo III	81.5	Progênies (THS 1188 x CCN 51)	Royaert et al. 2016
	Tcm004s00110232		Cromossomo IV	0.55		
	Tcm006s19715703		Cromossomo VI	31.01		
	Tcm006s25375496		Cromossomo VI	61.73		
	Tcm007s10302466		Cromossomo VII	47.55		
	Tcm009s02031341		Cromossomo IX	13.73		
	Tcm009s08066239		Cromossomo IX	44.36		
SSR	mTcCIR<br>SHRSTc<br>RGH<br>WRKY	Resistance WBD	NA	~2.6	Progênies de autofecundação de TSH 516	Brown et al. 2005
EST-SSR	mTcCIR 292	Defense-related protein	5′UTR	1.3	LAN28<br>SCA6<br>MAT 1–6<br>SA16<br>SNA1001<br>SNA1003<br>UPA402<br>UF676	Fouet et al. 2011
	mTcCIR 293	Pathogenesis-related protein	3′UTR			
	mTcCIR 294	Protein kinase	5′UTR			
	mTcCIR 297	Oleosin low molecular weight isoform	3′UTR			
	mTcCIR 298	Aspartyl protease family protein	3′UTR			
	mTcCIR 299	Transducin family protein wd-40 repeat family protein	5′UTR			
	mTcCIR 301	Leucine-rich receptor-like protein kinase	3′UTR			
	mTcCIR 302	Calcium-dependent protein kinase CP4	3′UTR			
	mTcCIR 303	Serine threonine protein	5′UTR			
	mTcCIR 304	7S vicilin	CDS			
	mTcCIR 305	Chloroplast acyl-acp thioesterase	5′UTR			

mTcCIR 306	3-ketoacyl-ACP synthase	5′UTR
mTcCIR 308	SNARE protein	5′UTR
mTcCIR 309	Glutelin	CDS
mTcCIR 310	24-sterol C-methyltransferase	5′UTR
mTcCIR 312	UDP-glucose pyrophosphorylase	5′UTR
mTcCIR 314	MADS-box protein	3′UTR
mTcCIR 315	Nucleic acid binding	3′UTR
mTcCIR 316	myb-like transcription factor 2	5′UTR
mTcCIR 317	Transcription factor IIa large subunit	5′UTR
mTcCIR 318	Floral homeotic protein apetala1	5′UTR
mTcCIR 319	bZIP transcription factor	5′UTR
mTcCIR 320	AP2/EREBP transcription factor	5′UTR
mTcCIR 322	Phantastica transcription factor A	5′UTR
mTcCIR 324	Scarecrow-like transcription factor 8	5′UTR
mTcCIR 325	Urease accessory protein G	5′UTR
mTcCIR 326	Phosphate phosphoenolpyruvate translocator-like protein	3′UTR
mTcCIR 327	Phosphoenolpyruvate carboxylase	5′UTR
mTcCIR 329	Lysine and histidine specific transporter protein	3′UTR
mTcCIR 331	Homeodomain-leucine zipper protein 56	5′UTR
mTcCIR 333	Nucleotide sugar	5′UTR

	epimerase-like protein	
mTcCIR 336	Plastid-lipid associated protein	CDS
mTcCIR 337	Fibrillin precursor-like protein	CDS
mTcCIR 339	GL1 protein	5′UTR
mTcCIR 341	Guanine nucleotide exchange factor P532	5′UTR
mTcCIR 342	3-methyl-2-oxobutanoate hydroxymethyltransferase	5′UTR
mTcCIR 343	Protein arginine N-methyltransferase family protein	5′UTR
mTcCIR 344	Late embryogenesis-abundant protein	5′UTR
mTcCIR 348	One-helix protein	CDS
mTcCIR 349	Arabinogalactan protein 2	3′UTR
mTcCIR 350	Unnamed protein product (G. hirsutum SSR)	CDS
mTcCIR 351	Lipoxygenase	3′UTR
mTcCIR 352	Unnamed protein product (G. hirsutum SSR)	5′UTR
mTcCIR 353	Progesterone-binding protein homolog	3′UTR
mTcCIR 354	Tyrosine specific protein phosphatase family protein	5′UTR
mTcCIR 355	Glycosyl transferase family 17 protein	5′UTR
mTcCIR 356	Auxin-induced protein IAA9	5′UTR
mTcCIR 358	Storage protein (late embryogenesis abundant).	CDS

mTcCIR 359	Amia calva GARS-AIRS-GART	3′UTR
mTcCIR 360	Phosphatidylserine synthase	5′UTR
mTcCIR 361	Nac domain protein	5′UTR
mTcCIR 363	Ubiquitin-conjugating enzyme	5′UTR
mTcCIR 364	Importin beta	5′UTR
mTcCIR 365	Cyclic nucleotide-gated calmodulin-binding ion channel	3′UTR
mTcCIR 366	Calcium-dependent protein kinase	5′UTR
mTcCIR 367	Gibberellin 3 beta-hydroxylase	5′UTR
mTcCIR 369	NAC2-like protein	5′UTR
mTcCIR 373	NAC domain protein	3′UTR
mTcCIR 374	Heat shock protein binding	5′UTR
mTcCIR 375	Immunophilin	5′UTR
mTcCIR 376	ABC transporter family protein	3′UTR
mTcCIR 378	Plastocyanin-like domain-containing protein	3′UTR
mTcCIR 379	RING-H2 finger protein RHG1a	5′UTR
mTcCIR 380	Glutathione reductase	5′UTR
mTcCIR 382	Protein disulfide isomerase	5′UTR
mTcCIR 383	Yippee-like protein	5′UTR
mTcCIR 384	RAN binding protein	5′UTR
mTcCIR 387	STI ATP binding DNA binding DNA-directed DNA polymerase	CDS
mTcCIR 388	3-phosphoinositide-dependent	5′UTR

	protein kinase-1	
mTcCIR 390	Villin 2	CDS
mTcCIR 391	heat shock protein	CDS
mTcCIR 392	NHL repeat-containing	5′UTR
mTcCIR 393	Inorganic pyrophosphatase	5′UTR
mTcCIR 394	Plastocyanin-like domain-containing protein	5′UTR
mTcCIR 397	Outer membrane OMP85 family protein	5′UTR
mTcCIR 398	Monocarboxylic acid transporters	CDS
mTcCIR 400	CALM_SPIOL calmodulin (CaM)	3′UTR
mTcCIR 402	MADS-box protein BM5A	3′UTR
mTcCIR 403	MADS-box interactor-like	CDS
mTcCIR 404	Transcription regulator	3′UTR
mTcCIR 405	Zinc finger protein OBP4	5′UTR
mTcCIR 406	Rab GTPase activator	3′UTR
mTcCIR 408	Zinc finger (C2H2 type) family protein	3′UTR
mTcCIR 409	Nucleoid DNA-binding protein CND41	5′UTR
mTcCIR 410	N-rich protein	5′UTR
mTcCIR 411	Somatic embryogenesis receptor kinase	3′UTR
mTcCIR 412	Sm-D1 protein	5′UTR
mTcCIR 413	Aldose 1-epimerase	5′UTR
mTcCIR 414	CCCH-type zinc finger transcription factor	CDS
mTcCIR 415	Glycosyl hydrolase family 1 protein	5′UTR

mTcCIR 416	AP2 domain containing protein	CDS
mTcCIR 418	Transcription factor	3′UTR
mTcCIR 419	Branched-chain amino acid aminotransferase	5′UTR
mTcCIR 420	Nucleic acid binding	5′UTR
mTcCIR 421	Mitotic checkpoint protein	5′UTR
mTcCIR 422	MYB transcription factor MYB92	5′UTR
mTcCIR 423	Receptor like protein	3′UTR
mTcCIR 424	CGS1 mRNA stability 1	3′UTR
mTcCIR 425	Serrate transcription factor	5′UTR
mTcCIR 426	ATP binding/ATP-dependent helicase	CDS
mTcCIR 428	Small heat shock protein	3′UTR
mTcCIR 429	Dihydroflavonol reductase	5′UTR
mTcCIR 430	Lipase-like protein	3′UTR
mTcCIR 431	Protein kinase	3′UTR
mTcCIR 432	Cytosolic factor	5′UTR
mTcCIR 433	Pathogen-induced calmodulin-binding protein	3′UTR
mTcCIR 434	Glyoxalase extradiol ring-cleavage dioxygenase	5′UTR
mTcCIR 436	Translation initiation factor (eIF-4A)	5′UTR
mTcCIR 438	Luminal binding protein	5′UTR
mTcCIR 439	WRKY family transcription factor	5′UTR

	mTcCIR 441	Nitrilase-associated protein	3′UTR			
	mTcCIR 444	Trypanothione-dependent peroxidase	5′UTR			
	mTcCIR 445	GTP-binding protein RAB11	5′UTR			
	mTcCIR 446	Heterogeneous nuclearribonucleoprotein A2	5′UTR			
	mTcCIR 447	NPH4 transcription factor	5′UTR			
	msEstTsh-1	DNA polimerase related	ND			
	msEstTsh-2	Expressed protein	5' UTR			
	msEstTsh-3	Protein F2D10.18	ORF	NA		
	msEstTsh-4	Expressed protein	ORF			
	msEstTsh-5	Unknown protein	ORF			
EST-SSR	msEstTsh-6	Unknown protein	ND			
	msEstTsh-7	Thyroid nuclear factor 1	ND			
	msEstTsh-8	Unknown protein	ORF		Clones Sca-6 Catongo	Lima et al. 2010
	msEstTsh-9	Unknown protein	ND			
	msEstTsh-10	Hypothetical protein	5' UTR			
	msEstTsh-11	Casein kinase	3' UTR			
	msEstTsh-12	Unknown protein	ORF			
	mTcCIR12					
	mTcCIR26					
	mTcCIR30					
	mTcCIR37					
SSR	mTcCIR58	NA	NA			
	mTcCIR157					
	mTcCIR166					
	mTcCIR215					
	mTcCIR251					
	mTcCIR35					
SSR	MTcCIR24					
	mTcCIR30	Resistance WBD	NA	NA	F2 (Sca-6 x ICS-1)	Faleiro et al. 2006
RAPD	agcat.78					
AFLP	AV14.940					

	SSR	mTcCIR91<br>mTcCIR183<br>TcSNP375<br>TcSNP720<br>TcSNP1230<br>TcSNP1374	Resistance WBD	Cromossomo IV, V, VI, VIII e X	NA	Acessos de cacau	Motilal et al. 2016
Theobroma grandiflorum	EST-SSR	c2723<br>c5718<br>c70<br>c180<br>c193B<br>c203B<br>c3202/3202B<br>c733<br>c339<br>c733<br>c431B	Resistance WBD	ORF<br>ORF<br>5' UTR<br>5' UTR<br>ORF<br>ORF<br>ORF<br>ORF<br>ORF<br>ORF<br>5' UTR	NA	Genótipos de cupuaçu	Dos Santos et al. 2016
	SNP	6M1252980	Resistance WBD	Cromossomo VI	1.7	Progênies (Clone 174 X Clone 1074)	Mournet et al. 2020

NA: not assigned
