# Supplementary material for: State of the Art of the Molecular Biology of the Interaction between Cocoa and Witches’ Broom Disease: A Systematic Review
Source: Int J Mol Sci. 2023 Mar 16;24(6):5684. doi: 10.3390/ijms24065684 (PMC10057015; doi:10.3390/ijms24065684)
Supplement: Supplementary file 1 [file ijms-24-05684-s001.zip › Supplementary Table S3.pdf]

Supplementary Table S3: Host proteins summarized from eligible studies in the systematic review.

Hospedeiro	Proteína	Função	Acúmulo	Órgãos	Autores
Theobroma cacao L.	TcCys1 (cystatin)	Defense, development of programmed cell death symptoms, inhibits mycelial growth and acts on hyphal shortening	↑	Leaves, meristems, roots, seeds and stems	Cascardo et al. 2010
	TcCys2 (cystatin)		↑		
	TcCys3 (cystatin)		↑		
	TcCys4 (cystatin)		↑		
Nicotiana benthamiana	60 kDa chaperonin subunit alpha (CPN-60 alpha)	NA	↑	Suspended cell	Villela-Dias et al. 2013
	Pyruvate decarboxylase (PDC)	oxidation-reduction	↑		
	UDP glucose 6 dehydrogenase (UDPG)	oxidation-reduction	↑		
	Enolase (ENO)	NA	↑		
	Alpha tubulin	NA	↑		
	Adenosylhomocys teinase	NA	↑		
	Malate dehydrogenase (MDH)	Citric acid cycle	↑		
	Heat shock protein 80	Stress response	↑		
	Annexin	Ligação ao Ca 2+	↑		
	Aconitate hydratase (ACO)	Citric acid cycle	↑		
	Elongation factor 2 (EF-2)	NA	↑		
	NADH ubiquinone oxidoreductase isoform 1 and 2	oxidation-reduction	↑		
	Superoxide dismutase Mn mitochondrial (MnSOD)	oxidation-reduction	↑		
	5-methyltetrahydrop teroyltriglutamate-	Methionine biosynthesis	↑		

homocysteine S-methyltransferase (MetE)		
Heat shock cognate 70 kDa	Stress response	↑
Alcohol dehydrogenase (ADH)	Oxidation-reduction	↑
Stromal 70 kDa heat shock	Stress response	↓
Nucleosome assembly protein 1 like protein 2	NA	↓
Glyceraldehyde 3 phosphate dehydrogenase (GAPDH)	NA	↓
Calreticulin	Protein folding	↓
2,3-bisphosphoglycerate-independent phosphoglycerate mutase (PGAM-i)	Glucose biosynthesis	↓
14-3-3-like protein GF14 epsilon	NA	↓
Glutathione S transferase (GST)	NA	↓
Copper chaperone	NA	↓
Glycine-rich RNA binding protein 3	NA	↓
Heat shock cognate 70 kDa protein 2	Stress response	↓
Heat shock protein 90 (HSP90)	Stress response	↓
Heat shock protein 81 (HSP81)	Stress response	↓
Fructose-bisphosphate aldolase cytoplasmic (aldolase)	Glucose biosynthesis	↓
Fructose bisphosphate	Glucose biosynthesis	↓

	aldolase like protein				
	l-ascorbate peroxidase 2 cytosolic (APX2)	Stress response	↓		
	Desiccation-related protein	Desiccation-related protein	NA		
	Peptide transporter PTR3	Peptide transporter	NA		
	RNAse_H	Degrades RNA hybrid RNA:DNA	NA		
	Protein p14	Pre-mRNA splicing	NA		
	Retrotransposon protein Ty1	Binds to DNA; related to disease resistance in plants	NA		
	NAC domain-containing protein	Transcription factor	NA		
	CRM family member 2	RNA splicing and ribosome maturation	NA		
	50S Ribosomal protein L4	Repressor of transcription or translation	NA		
Theobroma cacao (Genótipo resistente)	Pentatricopeptide repeat containing protein	Unknown function; may be involved in RNA processing and stabilization	NA	Filoplano	Almeida et al. 2017
	DNA-directed RNA polymerase 3	Synthesizes small rRNA and tRNA complex enzymes that are responsible for transcription of DNA into RNA	NA		
	50S ribosomal protein L7/L12	Ribonucleoprotein involved in protein translation	NA		
	RNAse_H (ribonuclease)	Cleaves RNA hybrid RNA:DNA	NA		
	β-Caryophyllene synthase	Isoprenoid biosynthesis (plant defense)	NA		
	Laccase 11	Oxidoreductase, process synthesis of lignin	NA		
	Transketolase chloroplastic	Participates in the Calvin cycle in photosynthesis (energy)	NA		

Leucine-rich repeat receptor kinase	Signal transduction; via signaling (receptor) in plant defense against pathogens	NA
E3 ubiquitin ligase PUB14	Proteins related to plant–pathogen interaction	NA
Retrotransposon/probable polyprotein	Binds to nucleic acids; disease resistance in plants (plant protection)	NA
Similar to pherophorin-like protein	Glycoprotein of the extracellular matrix (cell structure)	NA
α/β-Hydrolase domain-containing protein	Catalytic functions in lipid metabolism (metabolism)	NA
Transcription elongation factors IIS	Efficient formation or stability of the complex with RNA polymerase II (transcription)	NA
Serine carboxypeptidase S28 family protein	Protein degradation—Protease (plant defense)	NA
Terpene cyclase, C1 domain-containing protein	Isoprenoid biosynthesis (plant defense)	NA
Endonuclease/exonuclease/phosphatase	DNA repair	NA
RNA methyltransferase	Processing RNAs (transcription)	NA
Phosphatidylcholine-sterol O-acyltransferase	Metabolism of lipids, steroids; important in the process of leaf senescence	NA
B3 domain-containing protein REM16	Involved in regulation of transcription	NA
Armadillo repeat domain of β-catenin protein	Signal transduction in hormonal responses	NA
DNA repair protein Rad4 family	Participates in repairing damaged DNA nucleotide excision	NA
Glicoprotease 1	Protease	NA
Hypothetical protein	Unknown	NA

	O-Glycosyl hydrolases	Carbohydrate metabolism (metabolism)	NA		
	Protein 10 type rhomboid	Serine endoproteinase activity type—Serine protease	NA		
	Elongation factor G	Translocation of the ribosome during translation	NA		
	Chaperonin 60 kDa	Protein folding (plant defense)	NA		
	ATP synthase subunit α	Regulating the synthesis of ATP (energy)	NA		
	Elongation factor Tu	tRNA binds to the ribosome (translation)	NA		
	Elongation factor Ts	Phosphorylation of GDP to GTP (translation)	NA		
	Polyribonucleotide nucleotidyltransfer ase	Processing mRNA	NA		
	Outer membrane protein Omp38	Diffusion of small metabolites (cell structure)	NA		
	Phosphate-binding protein PstS	Transport of phosphate and response to stress (plant defense)	NA		
	ATP-dependent Clp protease proteolytic subunit	Degradation of proteins (plant defense)	NA		
	ATP synthase subunit β	Energy	NA		
Theobroma cacao L.	TcSBP	Proteína de resistência (Proteínas termoestável; se liga ao selenito e potencialmente pode ser capaz de se ligar a metais pesados).	NA	*In sílico*	Alves et al. 2019
Theobroma cacao L.	Tc -cAPX	Oxidative stress	NA	*In sílico*	Camillo et al. 2013
Theobroma cacao (Genótipo resistente)	Ascorbate peroxidase	Oxidative stress	↑	Ápices caulinares	Dos Santos et al. 2020
	Peroxidase superfamily protein	Oxidative stress	↑		
	Class III peroxidase	Oxidative stress	↓		

2-cysteine peroxiredoxin B	Oxidative stress	↓
Peroxidase 4	Oxidative stress	↓
Chaperonin CPN60 2 mitochondrial	Oxidative stress	↓
hypothetical protein CICLE_v10000948mg	Oxidative stress	↑
Glyceraldehyde-3-phosphate dehydrogenase C2 isoform 2	Oxidative stress	↑
Glyceraldehyde-3-phosphate dehydrogenase C2 isoform 1	Oxidative stress	↑
Cationic peroxidase 2 precursor	Oxidative stress	↑
Peroxidase superfamily protein isoform 1	Oxidative stress	↓
Superoxide dismutase	Oxidative stress	↑
Copper/zinc superoxide dismutase 2 isoform 1	Oxidative stress	↑
Peroxidase superfamily protein isoform 1	Oxidative stress	↓
Chlorophyll a-b binding protein 3, chloroplastic	Photosynthesis and carbohydrate metabolism	↓
Phosphomannomutase	Photosynthesis and carbohydrate metabolism	↓
6-phosphogluconate dehydrogenase family protein	Photosynthesis and carbohydrate metabolism	↑
Glyceraldehyde-3-phosphate dehydrogenase C2 isoform 1	Photosynthesis and carbohydrate metabolism	↑

Glyceraldehyde-3-phosphate dehydrogenase C2 isoform 2	Photosynthesis and carbohydrate metabolism	↑
Insulinase (Peptidase family M16) protein isoform 1	Photosynthesis and carbohydrate metabolism	↑
Photosystem I subunit D-2	Photosynthesis and carbohydrate metabolism	↓
Glycosyl hydrolase superfamily protein	Photosynthesis and carbohydrate metabolism	↑
Ribulose bisphosphate carboxylase/oxygenase activase 1 isoform 1	Photosynthesis and carbohydrate metabolism	↑
Aldolase superfamily protein isoform 1	Photosynthesis and carbohydrate metabolism	↑
Amidase family protein isoform 1	Photosynthesis and carbohydrate metabolism	↓
Light-harvesting chlorophyll B-binding protein 3	Photosynthesis and carbohydrate metabolism	↓
Lactate/malate dehydrogenase family protein	Photosynthesis and carbohydrate metabolism	↓
Phosphoglycerate kinase 1	Photosynthesis and carbohydrate metabolism	↑
Sedoheptulose-bisphosphatase	Photosynthesis and carbohydrate metabolism	↑
Glycosyl hydrolase family 38 protein isoform 1	Photosynthesis and carbohydrate metabolism	↑
Chlorophyll a-b binding protein, chloroplastic	Photosynthesis and carbohydrate metabolism	↓
hypothetical protein CICLE_v10012049mg	Photosynthesis and carbohydrate metabolism	↓

Putative uncharacterized protein	Photosynthesis and carbohydrate metabolism	↑
Putative Beta xylosidase alpha L arabinofuranosidase 2	Photosynthesis and carbohydrate metabolism	↑
putative miraculin-like protein 2	Stress and defense	↑
Voltage dependent anion channel 2	Stress and defense	↑
Heat shock protein 89.1 isoform 1	Stress and defense	↑
Adenine nucleotide alpha hydrolases-like superfamily protein	Stress and defense	↑
Chitinase A	Stress and defense	↑
21 kDa seed protein, putative	Stress and defense	↑
Mitochondrial HSO70 2 isoform 2	Stress and defense	↑
Prohibitin 2	Stress and defense	↑
MLP-like protein 28	Stress and defense	↓
Chloroplast heat shock protein 70 isoform 1	Stress and defense	↑
Carrot EP3–3 chitinase, putative isoform 1	Stress and defense	↑
Pathogenesis-related protein 10.5	Stress and defense	↑
Adenine nucleotide alpha hydrolases-like superfamily protein	Stress and defense	↑
2-cysteine peroxiredoxin B	Stress and defense	↓

	Pathogenesis-related protein P2 isoform 1	Stress and defense	↓
	Pathogenesis-related protein P2 isoform 2, partial	Stress and defense	↑
	Pathogenesis-related protein PR-4B	Stress and defense	↑
	Abscisic stress ripening protein	Stress and defense	↓
	21 kDa seed protein	Stress and defense	↓
	Osmotin 34	Stress and defense	↑
	17.6 kDa class II heat shock protein	Stress and defense	↑
	TCP-1/cpn60 chaperonin family protein	Stress and defense	↓
	class I chitinase	Stress and defense	↑
	Thaumatin-like protein	Stress and defense	↑
	Chaperonin CPN60 2 mitochondrial	Stress and defense	↓
	Ascorbate peroxidase	Oxidative stress	↑
	Peroxidase	Oxidative stress	↑
	Peroxidase 68	Oxidative stress	↑
	Class III peroxidase	Oxidative stress	↓
Theobroma cacao (Genótipo suceptível)	Peroxidase 4	Oxidative stress	↓
	Malate dehydrogenase cytoplasmic	Photosynthesis and carbohydrate metabolism	↑
	Sucrose synthase	Photosynthesis and carbohydrate metabolism	↑
	Pyrophosphate--fructose 6 phosphate 1 phosphotransferase subunit alpha	Photosynthesis and carbohydrate metabolism	↑

Rhamnose biosynthesis 1 isoform 1	Photosynthesis and carbohydrate metabolism	↑
hypothetical protein CICLE_v10032502mg	Photosynthesis and carbohydrate metabolism	↑
Malate dehydrogenase	Photosynthesis and carbohydrate metabolism	↓
PfkB-like carbohydrate kinase family protein	Photosynthesis and carbohydrate metabolism	↓
Beta-glucosidase 44	Photosynthesis and carbohydrate metabolism	↓
Enolase	Photosynthesis and carbohydrate metabolism	↓
NADP-dependent malic enzyme	Photosynthesis and carbohydrate metabolism	↓
PfkB-like carbohydrate kinase family protein	Photosynthesis and carbohydrate metabolism	↓
Aldolase superfamily protein isoform 1	Photosynthesis and carbohydrate metabolism	↓
Phosphoglycerate kinase cytosolic	Photosynthesis and carbohydrate metabolism	↓
Aldolase-type TIM barrel family protein isoform 1	Photosynthesis and carbohydrate metabolism	↑
Glucose-6-phosphate 1 dehydrogenase cytoplasmic isoform	Photosynthesis and carbohydrate metabolism	↑
Photosystem II subunit O-2	Photosynthesis and carbohydrate metabolism	↓
Methionine synthase	Photosynthesis and carbohydrate metabolism	↑
5-methyltetrahydropteroyltriglutamate--homocysteine methyltransferase	Photosynthesis and carbohydrate metabolism	↑

Prohibitin 2	Photosynthesis and carbohydrate metabolism	↑
Chloroplast heat shock protein 70 isoform 1	Photosynthesis and carbohydrate metabolism	↓
hypothetical protein CICLE_v1002798 1mg	Photosynthesis and carbohydrate metabolism	↑
heat shock protein 70B	Photosynthesis and carbohydrate metabolism	↑
Prohibitin 2	Photosynthesis and carbohydrate metabolism	↑
Osmotin 34	Photosynthesis and carbohydrate metabolism	↑
Basic chitinase	Photosynthesis and carbohydrate metabolism	↑
Glucan endo 1 3 beta glucosidase basic vacuolar isoform	Photosynthesis and carbohydrate metabolism	↑
Ankyrin repeat domain-containing protein 2 isoform 1	Photosynthesis and carbohydrate metabolism	↑
Uncharacterized protein TCM_004731	Photosynthesis and carbohydrate metabolism	↑
21 kDa seed protein	Photosynthesis and carbohydrate metabolism	↓
Voltage dependent anion channel 2	Photosynthesis and carbohydrate metabolism	↑
Prohibitin 3 isoform 1	Photosynthesis and carbohydrate metabolism	↓
MLP-like protein 28	Photosynthesis and carbohydrate metabolism	↓
Heat shock 70 kDa protein mitochondrial	Photosynthesis and carbohydrate metabolism	↓
Heat shock cognate protein 70–1	Photosynthesis and carbohydrate metabolism	↓
Heatshock cognate protein 80	Photosynthesis and carbohydrate metabolism	↓

	Acidic endochitinase	Photosynthesis and carbohydrate metabolism	↓		
	Putative miraculin-like protein 2	Photosynthesis and carbohydrate metabolism	↓		
Theobroma cacao L.	TcPHYLL (phylloplanin)	Pathogenicity (stress response)	NA	*In sílico*	Freire et al. 2017
Theobroma cacao L.	TcPR-1f (kinase domain)	Defense response	↑	Suspended cell	Tosarini et al. 2018
	TcPR-1g (kinase domain)	Defense response	↑		
Theobroma cacao L.	β-1,3-1,4-glucanase	Antifungal activity (Membrane hydrolysis)	↑	Suspended cell	Britto et al.2013
	TcLEG3 (Legumain)	Related to the seed	NA		
Theobroma cacao L.	TcLEG6 (Legumain)	Related with embryogenesis.	NA	*In sílico*	Santana et al. 2016
	TcLEG9 (Legumain)	Defense against pathogen	NA		
Theobroma cacao (Genótipo suceptível)	TcCYSPR04 (cysteine protease)	Defense response	↑	Fluido apoplástico	Cardoso et al. 2015
Theobroma cacao (Genótipo resistente)	TcCYSPR04 (cysteine protease)	Defense response	↑		
Theobroma cacao L.	Proteína de ligação BiP	Molecular Chaperone	↑	Meristema	Alvim et al. 2009
Theobroma cacao L.	TcPR-4b	Antifungal activity; RNase and DNase activity	NA	*In sílico*	Menezes et al. 2014
Theobroma cacao L.	TcWRKY	Transcription Factor	NA	*In sílico*	De Almeida et al. 2017
Theobroma cacao (Genótipo suceptível)	TcTI (Trypsin Inhibitor)	Antifungal activity (inhibitory action)	↑	Meristema	Amaral et al. 2022
Theobroma cacao (Genótipo resistente)	TcTI (Trypsin Inhibitor)	Antifungal activity (inhibitory action)	↓		
Theobroma cacao L.	TcOsm1	Antifungal activity (antimicrobial action, inhibition of mycelial growth)	↑	Suspended cell	Falcao et al. 2016
	Osm-pepA	Antifungal activity (antimicrobial action,	↑		

		inhibition of mycelial growth)			
	Osm-pepB	Antifungal activity (antimicrobial action, inhibition of mycelial growth)	↑		
Theobroma cacao L.	TcPR-10	Antifungal activity (Action as ribonuclease and allergenic potential)	↑	Suspended cell	Menezes et al. 2021

NA: not assigned
