# Supplementary material for: State of the Art of the Molecular Biology of the Interaction between Cocoa and Witches’ Broom Disease: A Systematic Review
Source: Int J Mol Sci. 2023 Mar 16;24(6):5684. doi: 10.3390/ijms24065684 (PMC10057015; doi:10.3390/ijms24065684)
Supplement: Supplementary file 1 [file ijms-24-05684-s001.zip › Supplementary Table S4.pdf]

Supplementary Table S4: Pathogen proteins summarized from eligible studies in the systematic review.

d **Tabela X**: Proteínas do fungo (legenda a melhorar)

Protein	Function	Accumulation	Region	Authors
MpNEP1	Induction of necrosis and ethylene synthesis	↑	Biotrophic and saprotrophic mycelium	Garcia et al. 2007
MpNEP2	Induction of necrosis and ethylene synthesis	↑		
Aldo-keto reductase	Stress response	↑	Mycelium	Pierre et al. 2017
Malate dehydrogenase	Metabolism enzyme	↑		
Phosphoglycerate kinase	Metabolism enzyme	↑		
NAD-dependent epimerase/dehydratase	Metabolism enzyme	↑		
1-Cys peroxiredoxin	Peroxide decomposers	↑		
Acetyl-acetyltransferase	NA	↑		
Aspartate aminotransferase	NA	↑		
Pleurotolysin B homologue	NA	↑		
Erylysin B	NA	↑		
Glyceraldehyde-3-phosphate dehydrogenase	Metabolism enzyme	↑		
Heat-shock protein hss1	Stress response	↑		
Enolase	Metabolism enzyme	↑		

Nitrilase	Auxin biosynthesis and cyanide detoxification	↑		
Mannitol-1-phosphate dehydrogenase	Mannitol biosynthesis	↑		
MPER_00772	Unknown function	↑		
NAD-dependent formate dehydrogenase	Methanol catabolism	↑		
Putative anhydrolase	NA	↑		
ATP binding cassete	Fungal pathogenicity	↑		
PR-1	Fungal pathogenicity	↑		
Initial translation factor	Metabolism and energy	↓		
Nucleoside diphosphate kinase	Metabolism and energy	↓		
Small ribosomal subunit	Metabolism and energy	↓		
60S ribosomal protein	Metabolism and energy	↓		
Serine protease inhibitor	Metabolism and energy	↓		
Nucleoside triphosphate hydrolase	Metabolism and energy	↑		
Large ribosomal subunit	Metabolism and energy	↑	Basidiospores	Mares et al. 2017
Translation elongation factor 1α	Metabolism and energy	↑		
Folding protein	Metabolism and energy	↑		
Heat shock protein (HSP70)	Metabolism and energy	↑		
Binding immunoglobulin protein (BiP)	Metabolism and energy	↑		
ATP synthase	Metabolism and energy	↑		
Glyceraldehyde-3-phosphate dehydrogenase	Metabolism and energy	↓		

Valosin contain protein (VCP)	Cell cycle regulation	↑		
Kinesin	Cell cycle regulation	↑		
Septin	Primary hypha septation	↑		
Oxidorredutase	Stress response and anti-oxidation	↑		
Ascorbate peroxidase	Stress response and anti-oxidation	↑		
Catalase	Stress response and anti-oxidation	↑		
Superoxide dismutase	Stress response and anti-oxidation	↑		
Peptidyl-prolyl cis-trans isomerase	Fungal pathogenicity	↑		
Transcription factor FapR	Fungal pathogenicity	↑		
Polyketide synthase	Fungal pathogenicity	↑		
MpPR-1	Neutralization of plant defenses, antimicrobial activity to avoid competitors and fruiting body physiology	NA	Dikaryotic mycelium	Teixeira et al. 2012
MpAtg8p	Autophagy	NA	*In sílico*	Pereira et al. 2013
Reverse transcriptase (RT-Like)	Transposition of transposable elements	↑	NA	Pereira et al. 2007
Acyl-CoA binding protein (ACBP)	Intracellular acyl-CoA transporter	↑	*In sílico*	Monzani et al. 2010
Glyceraldehyde-3-phosphate dehydrogenase (MpGAPDH)	Fungal pathogenicity and glucose biosynthesis	↑	*In sílico*	Lima et al. 2009

Lanosterol 14α-demethylase (ERG11)	Ergosterol biosynthesis	↑	Mycelium	Ceita et al. 2014
Putative effector	Catabolic process	DA		
Putative effector	Cell wall organization or biogenesis	DA		
Putative effector	Cellular metabolic process	DA		
Putative effector	Cellular process	DA		
Putative effector	Establishment of localization	DA		
Putative effector	Localization, single-organism process	DA		
Putative effector	Metabolic process	DA		
Putative effector	Multi-organism process	DA		
Putative effector	Nitrogen compound metabolic process	DA	Mycelium	Barbosa et al. 2018
Putative effector	Organic substance metabolic process	DA		
Putative effector	Pathogenesis	DA		
Putative effector	Primary metabolic process	DA		
Putative effector	Response to stress	DA		
Putative effector	Single-organism cellular process	DA		
Putative effector	Single-organism metabolic process	DA		
Putative effector	Carbohydrate binding	DA		

Putative effector	Heterocyclic compound binding	DA		
Putative effector	Hydrolase activity	DA		
Putative effector	Ion binding	DA		
Putative effector	Lyase activity	DA		
Putative effector	Organic cyclic compound binding	DA		
Putative effector	Oxidoreductase activity	DA		
Putative effector	Pattern binding	DA		
Putative effector	Peroxidase activity	DA		
Putative effector	Structural constituent of cell wall	DA		
Putative effector	Cell periphery	DA		
Putative effector	External encapsulating structure	DA		
Putative effector	Intrinsic component of membrane	DA		
Metal-dependent hydrolase (MDH)	Fungal pathogenicity	NA		
Necrosis-inducing proteins (NEPs)	Fungal pathogenicity	NA	NA	Tibúrcio et al. 2009
Mannitol 1-phosphate 5-dehydrogenase(MPDH)	Fungal pathogenicity	NA		
Thaumatin-like proteins (TLPs)	Fungal pathogenicity	NA	Basidiospores	Franco et al. 2015
Cerato-platanin (CP)	Necrosis inducing	NA	Saprotrophic mycelium	Zaparoli et al. 2009

Pyridoxal-dependent decarboxylase	Indoleacetic acid metabolism and synthesis (AIA)	↑		
Decarboxyl L-amino acids	Indoleacetic acid metabolism and synthesis (AIA)	↑		
Nitrilase	Indoleacetic acid metabolism and synthesis (AIA)	↑		
NAD-dependent aldehyde dehydrogenase	Indoleacetic acid metabolism and synthesis (AIA)	↑		
RAB GDP dissociation inhibitor	Cell proliferation	↑		
homocysteine methylase (HCM)	Methionine metabolism	↑		
Alanine dehydrogenase	Pigment biosynthesis	↑		
Hypothetical protein (gi|238614130)	Possible specificity for fungal division	↑		
GDI	Regulation	↓	Necrotrophic mycelium and basiodiocarps	Gomes et al. 2021
SEC4	Signaling processes/regulation of vesicle transport and autophagy	↑		
RAS	Signaling processes/regulation of vesicle transport and autophagy	↑		
Ciclophyilin	Protein folding and trafficking, apoptosis, signal reception, alteration of gene expression, ROS detoxification	↑		
Glycine-rich RNA binding protein 1 (GRP1)	Stress response	↑		
Ciclophyilin (CYT450)	Stress response	↑		

Agglutinins	Response to stress and nutrient deficiency	↑		
Glyceraldehyde-3-P dehydrogenase (GAPDH)	Glucose biosynthesis	↑		
aldo-keto reductase (AKR)	Stress response	↑		
Tyrosine phosphatases (TYP)	Osmotic and oxidative stress response	↑		
Linoleate diol synthase	Lipid biosynthesis	↑		
Leukotriene-A4 hydrolase	Lipid biosynthesis	↑		
3-ketoacyl-coA-thiolase	Lipid biosynthesis	↑		
Phosphatidylserine decarboxylase (PSD)	Phospholipid synthesis	↑		
Δ-1-pyrroline-5-carboxylate dehydrogenase (P5Cdh	Sporulation and virulence	↑		
N-acetyl-gamma-glutamyl phosphate reductase(ARGC)	Synthesis of ornithine precursors	↑		
Arginase	Synthesis of ornithine precursors	↑		
Rubber elongation factor protein (REF)	Latex biosynthesis	↑		
Heat shock proteins (HSP)	Protein synthesis and folding, transport of proteins across the membrane, protein disassembly and protein degradation	↑		
pleurotolysin PriA	Basidiocarp formation	↑		
ATP synthase	Energy metabolism	↑	Basidiospores	Mares et al. 2020

Binding immunoglobulin protein (BiP)	Energy metabolism	↑		
alcohol dehydrogenase (ADH)	Energy metabolism	↑		
Polyketide cyclase	Fungal pathogenicity	↑		
Glycoside hydrolase	Carbohydrate metabolism	↑		
Major facilitator superfamily (MFS)	Resistance	↑		
Catalase A	Anti-oxidation	↑		
Cerato-platanins (MpCP1)	Fungus-host interaction (hyphae growth, fruiting body formation and substrate adhesion)	DA		
Cerato-platanins (MpCP2)	Fungus-host interaction (hyphae growth, fruiting body formation and substrate adhesion)	DA		
Cerato-platanins (MpCP3)	Fungus-host interaction (hyphae growth, fruiting body formation and substrate adhesion)	DA	Basidiospores	Barsottini et al. 2013
Cerato-platanins (MpCP4)	Fungus-host interaction (hyphae growth, fruiting body formation and substrate adhesion)	DA		
Cerato-platanins (MpCP5)	Fungus-host interaction (hyphae growth, fruiting body formation and substrate adhesion)	DA		
MpPR-1 (pathogenesis-related 1)	Fungal pathogenicity (lipid transport)	↑	NA	Darwiche et al. 2017

Glyceraldehyde-3-phosphate dehydrogenase	Oxidoreductase	↑		
Heat shock protein HSS1	Stress response	↑		
Eukaryotic translation initiation factor 5A-1	Protein biosynthesis	↑		
14-3-3 protein homolog	Stress response	↑		
ATP synthase subunit beta	Transport	↑		
Protein disulfide-isomerase 1	Cell redox homeostasis	↑		
	Stress response			
78-kDa glucose-regulated protein homolog		↑		
Cobalamin synthase	Zinc ion binding	↑		
			Hypha	Silva et al. 2013
Thiazole synthase	Suicide enzyme; stress response and in DNA damage tolerance	↑		
Malate dehydrogenase	Oxidoreductase	↑		
Acetyl-CoA acetyltransferase	Ergosterol biosynthetic process	↑		
ATP synthase subunit alpha, mitochondrial	ATP catabolic process	↑		
Phosphoglycerate kinase	Phosphoprotein	↑		
S-adenosylmethionine synthase 2	One-carbon metabolism	↑		
Glyceraldehyde-3-phosphate dehydrogenase	Oxidoreductase	↑		
Chaperone protein DnaK	Stress response	↑		

ATP-dependent protease ATPase subunit HslU (heat shock)	Stress response	↑
Pyruvate kinase	Carbohydrate metabolism	↑
Autophagy-related protein 18	Autophagy	↑
Ubiquitin-conjugating enzyme	Postreplication repair	↑
Transaldolase	Pentose-phosphate shunt (stress response)	↑
Probable phosphoketolase	Stress response	↓
Glyceraldehyde-3-phosphate dehydrogenase	Oxidoreductase	↓
GTP-binding nuclear protein	Autophagy	↓
Dihydroxy-acid dehydratase, mitochondrial	Amino acid biosynthesis	↓
Cell division control protein 48	Cell cycle; Autophagy	↓
60S ribosomal protein L6	Cytoplasmic translation	↓
Malate dehydrogenase, mitochondrial	Oxidoreductase	↓
Catalase	Oxidoreductase	↓
6-Phosphogluconate dehydrogenase,decarboxylating 1	Oxidoreductase	↓
5-Methyltetrahydropteroyltriglutamate	Stress response	↓
Chaperone protein ClpB	Stress response	↓
Heat shock protein 82	Stress response	↓
Heat shock protein 90-2	Stress response	↓
Heat shock protein HSS1	Stress response	↓

Heat shock cognate 90-kDa protein	Stress response	↓
Heat shock protein Hsp88	Stress response	↓
Thiazole synthase	Stress response	↓
40S ribosomal protein	Ribosome biogenesis	↓
Nucleoside diphosphate kinase	Response to DNA damage stimulus	↓
Nucleoside diphosphate kinase Ndk1	Repair of UV radiation- and etoposide-induced DNA damage	↓
Proteasome component PRE6	Proteasomal ubiquitin-dependent protein catabolic process; regulation of mitotic cell cycle	↓
UPF0107 protein TSIB_1943	Phosphorylation	↓
Alanine--tRNA ligase, mitochondrial	Protein biosynthesis	↓
V-type proton ATPase subunit B	Phosphoprotein	↓
Mitochondrial-processing peptidase subunit beta	Phosphoprotein	↓
Aspartyl/glutamyl-tRNA(Asn/Gln) amidotransferase subunit B	Protein biosynthesis	↓
ATP-dependent RNA helicase fal1	rRNA processing	↓
Adenosylhomocysteinase	Phosphoprotein	↓
Asparagine synthetase [glutamine-hydrolyzing] 1	Phosphoprotein	↓
SWI5-dependent HO expression protein 3	Transport	↓

Paxillin-B	Zinc ion binding	↓		
*RPS1	Protein synthesis or degradation			
*UM00868.1	Protein synthesis or degradation			
* UM05990.1	Protein synthesis or degradation			
* RPS0	Protein synthesis or degradation			
* UM03578.1	Protein synthesis or degradation			
* UM01318.1	Protein synthesis or degradation			
* UM04986.1	Protein synthesis or degradation	NA	Non-germinated basidiospores	Mares et al. 2016
* UM04971.1	Cellular metabolism			
* UM04562.1	Cellular metabolism			
* UM02461.1	Cellular metabolism			
* UM00595.1	Cellular metabolism			
* UM02562.1	Cellular metabolism			
* Tef1	Cellular metabolism			
* UM05993.1	Mitochondrial protein			
* UM05090.1	Ion transport			
* UM04871.1	Ion transport			

* UM03951.1	Ion transport
* UM00621.1	Ion transport
* UM03356.1	Ion transport
* UM03527.1	Ion transport
* UM04971.1	Ion transport
* UM04562 .1	Ion transport
* UM00595.1	Ion transport
* UM02462.1	Ion transport
* UM01672.1	Cytoskeleton regulation and organization
* UM0068.1	Cytoskeleton regulation and organization
* UM00403.1	Cytoskeleton regulation and organization
* UM05379.1	Cytoskeleton regulation and organization
* UM05918.1	Cytoskeleton regulation and organization
* UM06453.1	Cytoskeleton regulation and organization
* UM04507.1	Cytoskeleton regulation and organization
* UM02715.1	Sporulation, reproduction and cell differentiation

* UM028991.1	Sporulation, reproduction and cell differentiation
* UM03449.1	Sporulation, reproduction and cell differentiation
* UM03734.1	Sporulation, reproduction and cell differentiation
* UM06217.1	Sporulation, reproduction and cell differentiation
* CDC42	Sporulation, reproduction and cell differentiation
*UAC1	Sporulation, reproduction and cell differentiation
* KPP6	Sporulation, reproduction and cell differentiation
* RPB1	Transcription
* UM03988.1	Transcription
* UM00157.1	Pyruvate kinase (transcription)
* UM02776.1	Nucleoside diphosphate kinase (transcription)
* UM06156.1	Transcription
* UM06331.1	Transcription
* UM05334.1	Transcription
* UM03058.1	Transcription
* UM02324.1	Transcription
* UM04722.1	Transcription

* UM02903.1	Transcription

NA: not assigned
DA: differentially accumulated em diferentes condições experimentais dos estudos analisados.
*Orthologous proteins of *Ustilagos maydis* in *Moniliophthora perniciosa* through System Biology.
