# Supplementary material for: State of the Art of the Molecular Biology of the Interaction between Cocoa and Witches’ Broom Disease: A Systematic Review
Source: Int J Mol Sci. 2023 Mar 16;24(6):5684. doi: 10.3390/ijms24065684 (PMC10057015; doi:10.3390/ijms24065684)
Supplement: Supplementary file 1 [file ijms-24-05684-s001.zip › Systematic Review Protocol.pdf]

Systematic Review Protocol

**Title**	The state of the art of the molecular biology of the interaction between cocoa and witches' broom disease
**Researchers**	Ariana Silva Santos<br>Irma Yuliana Mora Ocampo<br>Diogo Pereira Silva de Novais<br>Carlos Priminho Pirovani
**Description**	Witches' broom disease (WBD) caused by the hemibiotrophic fungus *Moniliophthora perniciosa* is one of the most important socio-economic phytosanitary problems of cocoa crops in the Americas. Scientific advances towards the elucidation of this pathosystem have occurred in recent years, but the Molecular Biology understanding of this pathogen-host interaction is still a field with many unanswered questions. In order to gain new insights and understand WBD at a molecular level, we present the first systematic review on the topic. Scopus, Web of Science, Pubmed and Scielo databases were used. A total of 1118 studies were extracted from the databases. Of these, 109 were eligible for data summarization and to answer the scientific questions of the review, using the inclusion and exclusion criteria, based on the Prisma guidelines. Eligible studies show that understanding the transition from the biotrophic-necrotrophic phase of the fungus is crucial for disease control. In recent years, protein profiles of *M. perniciosa* have been traced and some of the proteins have great biotechnological potential or can be targets for intervention, but tests in this regard are still lacking. Eligible articles in this study also revealed the potential genes in the interaction of *M. perniciosa* and hosts and Molecular Markers more efficient in the search for genetic variability and source of resistance. We highlight an arsenal of effectors already identified and not explored in the *M. perniciosa* x hosts pathosystem. This Systematic Review contributes to the understanding of the Molecular Biology of *M. perniciosa* and its interaction with the hosts, in addition, it offers new insights in this field of study and proposes different paths for the development of new strategies to control WBD.
**Objective**	To demonstrate the state of the art of the molecular biology of witches' broom disease caused by *M. perniciosa*

**Main questions**	Which are the main research groups that study the witches' broom disease caused by *M. perniciosa*?<br><br>Which countries carry out research on witches' broom disease caused by *M. perniciosa*?<br><br>Which are the areas of knowledge of publications on witches' broom disease caused by *M. perniciosa*?<br><br>Which are the hosts of the fungus M. perniciosa and the frequency of publications by hosts? are the main research groups that study the witches' broom disease caused by *M. perniciosa*?<br><br>Which countries carry out research on witches' broom disease caused by *M. perniciosa*?<br><br>Which are the areas of knowledge of publications on witches' broom disease caused by *M. perniciosa*?<br><br>Which are the hosts of the fungus *M. perniciosa* and the frequency of publications by hosts?<br><br>Which are the molecular mechanisms induced in the *M. perniciosa* fungus in the infection process or in its development?<br><br>Which are the molecular mechanisms induced in the hosts in the infection process caused by *M. perniciosa*?<br><br>Which are the genes related to resistance (or susceptibility) in the interaction between *M. perniciosa* and its hosts?<br><br>Which epigenetic mechanisms are involved in host resistance or susceptibility?<br><br>Which genes are involved in the pathogenicity (virulence) of the fungus?<br><br>Which are the molecular markers associated with host resistance?<br><br>Which sources of resistance have been developed against the fungus *M. perniciosa*?

	Which morphological changes does the *M. perniciosa* fungus undergo in order to succeed in the infection process?<br><br>Which morphological changes do the hosts undergo in the process of infection caused by the fungus *M. perniciosa*?<br><br>Which genes and proteins are involved in the molecular battle of *M.perniciosa* x host interaction?

**Keywords**	Systemetic review, Theobroma; witch's broom, molecular interaction
**Search strings**	((theobroma OR cacao OR cocoa OR moniliophthora OR crinipellis) AND (perniciosa OR broom))
**Search source selection criteria**	Scientific articles indexed in peer review journals.
**Research method**	Use of "search strings" and programs to help organize data.
**Database for research**	Pubmed, Scopus, Scielo and Web Of Science.
**Inclusion criteria**	- Primary articles;<br>- Articles in English, Spanish and Portuguese;<br>- Studies on the molecular biology of the interaction *M.perniciosa* and its hosts;<br>- Articles that are in line with the objective of the systematic review.
**Exclusion Criteria**	- Studies only with agroforestry/cabrucas systems<br>-Only morphological studies on M. perniciosa and/or host<br>- Taxonomic, anatomical, phylogenetic and ecological studies on M.perniciosa and/or hosts<br>- Review articles;<br>- Manuals<br>- Technical reports;<br>- Book chapters;<br>- Theses and dissertations;<br>- Abstracts;<br>- Articles published in annals of events.<br>- Articles that are not aligned with the objective of the systematic review.
**Definition of types of studies**	Based on inclusion and exclusion criteria.
**Evaluation of the quality of the studies**	Based on the methods, experimental design and results of the studies.
**Initial selection of studies**	Reading titles and abstracts.
**Final selection of studies**	- Full paper;<br>- Present the inclusion criteria;<br>- Not presenting one of the exclusion criteria.

**Data Extraction Strategy**	- Research groups/Research Centers/Laboratories and/or Universities that develop the studies;<br>- Study countries;<br>- Area of knowledge of publications;<br>-Hosts of the fungus;<br>-Molecular mechanisms induced in the fungus;<br>-Molecular mechanisms induced in the hosts;<br>- Genes related to resistance or susceptibility (hosts);<br>- Genes related to virulence (fungus);<br>- Sources of resistance developed (hosts);<br>- Molecular resistance markers (host);<br>- Genes expressed in the fungus x hosts interaction;<br>- Proteins accumulated in the fungus x hosts interaction;<br>- Biological function of genes and/or proteins expressed in the fungus x hosts interaction;<br>- Epigenetic mechanisms involved in resistance or susceptibility (hosts);<br>- Morphological changes in the fungus in the infection;<br>- Morphological changes of hosts when infected.
**Data summarization**	Graphs, tables and figures.
